# Promiscuous Receptors and Neuroinflammation: The Formyl Peptide Class

**DOI:** 10.3390/life12122009

**Published:** 2022-12-02

**Authors:** Edward S. Wickstead, Egle Solito, Simon McArthur

**Affiliations:** 1 Department of Neurology, Friedman Brain Institute, Icahn School of Medicine at Mount Sinai, New York, NY 10029, USA; 2 William Harvey Research Institute, Barts and The London School of Medicine and Dentistry, Queen Mary University of London, London E1 2AT, UK; 3 Department of Medicina Molecolare e Biotecnologie Mediche, University of Naples “Federico II”, 80131 Naples, Italy; 4 Institute of Dentistry, Faculty of Medicine & Dentistry, Queen Mary University of London, Blizard Institute, 4, Newark Street, London E1 2AT, UK

**Keywords:** formyl peptide receptors, FPR1, FPR2, GPCR, neuroinflammation, neurodegeneration

## Abstract

Formyl peptide receptors, abbreviated as FPRs in humans, are G-protein coupled receptors (GPCRs) mainly found in mammalian leukocytes. However, they are also expressed in cell types crucial for homeostatic brain regulation, including microglia and blood–brain barrier endothelial cells. Thus, the roles of these immune-associated receptors are extensive, from governing cellular adhesion and directed migration through chemotaxis, to granule release and superoxide formation, to phagocytosis and efferocytosis. In this review, we will describe the similarities and differences between the two principal pro-inflammatory and anti-inflammatory FPRs, FPR1 and FPR2, and the evidence for their importance in the development of neuroinflammatory disease, alongside their potential as therapeutic targets.

## 1. Introduction

The formyl peptide receptors (FPRs) are seven-pass, transmembrane G-protein coupled receptors (GPCRs) crucially involved in the inflammatory response. Although their roles in the response to infection and sterile peripheral inflammation have been extensively studied, their function in the central nervous system (CNS) and neuroinflammatory responses has only gradually become apparent [1,2,3,4,5]. In this review, we detail the similarities and differences between the two primary FPR family members, FPR1 and FPR2, and their immunological functions. We will focus on the growing evidence that these receptors play a key role in neuroinflammation. Through considering this evidence, we will make the case that these receptors have great potential as novel targets for therapeutic intervention in neuroinflammation, offering new ways to treat some of the most intractable of human diseases.

## 2. Inflammation

Inflammation is a complex biological process essential in responding to both tissue injury and infection. Ideally, it is a protective process, including the involvement of both cells from the immune system and the vascular endothelium, as well as a vast array of molecular mediators [6]. It serves to eliminate the initial cause of cell injury, help remove damaged tissue, and stimulate repair mechanisms.

One of the primary triggers of inflammation is infection, wherein an acute insult activates both the initial innate and later adaptive arms of the immune system. The initial innate response, characterized by a broad and non-specific pro-inflammatory signaling cascade, involves the release of inflammatory mediators including cytokines, chemokines, and reactive oxygen species (ROS). This response facilitates the activation and movement of immune cells such as neutrophils and monocytes in the periphery or microglia in the CNS towards the injury site, wherein pathogen killing commences. The innate immune system can also activate a more precise adaptive response, wherein T and B cells undergo clonal selection, responding to a specific antigen. Their activation triggers the engagement and specific targeting of both adaptive and innate effectors, including natural killer cells and neutrophils [7]. This dualistic approach provides acute protection but also extended surveillance from future, repeated pathogenic exposure. Sterile inflammation, a response characterized not by microorganisms but by insults such as mechanical trauma, toxins, and chemicals, is primarily associated with activation of innate immunity without vast adaptive input.

Following insult removal, innate cells transition towards pro-resolving phenotypes, which are responsible for the degradation of cellular debris and apoptotic cells, including effete immune cells, in tandem with supporting tissue repair. Upon successful transition, inflammation will begin to subside. However, if this transition fails, chronic inflammation can result—a response associated with the development of many human diseases [8,9,10].

## 3. The Formyl Peptide Receptors

The formyl peptide receptors, abbreviated as FPRs in humans, are pattern recognition receptors (PRRs) with central roles in host defense and inflammation [1,11,12,13]. Although expressed in a number of different cell types, the actions of FPRs have primarily been investigated in cells of myeloid origin; human FPR1 and FPR2 were originally identified in neutrophils and monocytes, while FPR3 was only detected in the latter [14]. These receptors have a diverse array of functions, from eliciting cellular adhesion and directed migration of recruited immune cells through chemotaxis, to granule release and superoxide formation [12,15,16]. The importance of these receptors in non-myeloid cell types has been reported more recently [17,18,19].

This receptor class was initially identified and named based on their ability to bind *N*-formylated peptides such as *N*-formylmethionine (fMet), produced through the degradation of both bacteria and mitochondria [20,21]. The ability to recognize *N*-formyl peptides, including the potent FPR1 agonist and chemotactic agent *N*-formyl-methionyl-leucyl-phenylalanine (fMLF), led to the conclusion that FPRs act as PRRs [3,22]. Following their original description, accumulating evidence has shown FPRs to bind to a diverse and continually expanding repertoire of ligands, including not only *N*-formyl peptides, but also non-formyl peptides of both microbial and host origin, synthetic small molecules, and eicosanoid lipids (Table 1). These molecules all bind to one or several FPRs and have been reviewed in detail previously [2,12,23].

There are three genes which encode for human FPRs: *FPR1*, *FPR2*, and *FPR3*. All three proteins share similar sequence homology and are encoded by genes clustered together on chromosome 19q13.3 in the human genome (Gao et al., 1998; Yi et al., 2007). Of these receptors, *FPR1* and *FPR2* share a particularly high overall gene sequence homology, with some overlapping functionality [2]. Comparatively, the genes which encode FPRs vary considerably in number between different species. For example, mice have eight known members of the *FPR* gene family on chromosome 17A3.2, denoted as ‘*Fprs*’. Despite the discrepancy in receptor numbers across the two species, several receptors share similar functionality, including FPR1/Fpr1, both of which are known to respond to host infection [59] and regulate chemotaxis [15,60,61]. These similarities extend to human *FPR2* as well, although murine functionality is encoded by two receptors which work in synergy to carry out comparable functions: *Fpr2/3* [62,63]. Highlighting their parallels, amino acid BLAST alignment confirms that these murine receptors display 76% (Fpr2) and 74% (Fpr3) identity alongside 85% (Fpr2) and 81% (Fpr3) homology to human FPR2, while Fpr2 and Fpr3 display 82% identity and 88% homology to each other.

For many years, the crystalline structure of these receptors remained elusive. Instead, structure simulation and molecular modeling [64], computer-aided ligand docking [65,66] and site-directed mutagenesis [67,68] had led to the identification of amino acids within both FPR1 and FPR2 responsible for receptor interactions with several different molecules [12]. More recently, the crystalline structure for FPR1 bound to the pan-formyl-peptide agonist fMLFII was reported with a resolution of 3.2 Å [69]. Further, two independent research groups reported crystalline structures for human FPR2 bound to the hexapeptide WKYMVm—a strong agonist for the receptor—with resolutions of 2.8 Å and 3.17 Å, respectively [13,70]. Zhaung and colleagues expanded further on the crystalline structure of FPR2, reporting interactions with several other known receptor agonists, fMLFII, the anti-inflammatory peptide CGEN-855A, and the synthetic anti-inflammatory small molecule Compound-43 with 3.1, 2.9 and 3.0 Å resolution, respectively [69]. Interestingly, structural comparison of these receptor-agonist conformations indicate the presence of a conserved receptor activation mechanism, suggesting that despite the ligands’ structural differences, receptor stimulation occurs due to similar molecular interactions. However, while these studies provide novel insights into the binding mechanisms of different ligands, it is crucial that the development of FPR crystalline structures continues, including interactions with receptor antagonists like cyclosporin H and WRW_4_, alongside pathogenic ligands such as serum amyloid A and β-amyloid (Aβ). In terms of the latter, cryo-electron microscopy recently helped elucidate the interaction between Aβ_1-42_ and FPR2 [71]. However, follow-up research will be important to decipher whether different Aβ formulations—such as monomers, oligomers, or fibrils—display different binding characteristics with this promiscuous receptor.

In summary, identification of novel receptor binding pockets for both pro-resolution and disease associated ligands may prove crucial for the future development of improved FPR associated therapeutics.

## 4. Cellular Expression of FPRs in the Central Nervous System

In contrast to their extensive analysis in the periphery, the role of these receptors in the CNS has only recently begun to be addressed [72,73,74]. In tandem with many research studies [1,75,76,77,78], proteome (Allen Brain Atlas) and transcriptome (Human Brain Transcriptome Project) datasets report FPR family expression within both human and rodent microglia, though the expression profile of FPRs within the brain extend further. Both Fpr1 and Fpr2 are expressed in mouse and rat neuronal stem cells, [79,80,81], and murine endothelial cells [17,82]. There is some evidence supporting neuronal Fpr2 expression in the spinal cord, hippocampus, prefrontal cortex and cerebellum of adult rats [83] alongside murine dorsal root ganglia [84] and in murine neuroblastoma cells [72]. However, evidence supporting similar expression profiles for Fpr1 in these cell types remain limited. Finally, while several studies report FPR2 expression in astrocytes [41,85], there are also more recent conflicting reports [83,86]. Unfortunately, the expression patterns of FPR1/Fpr1 and FPR3/Fpr3 need to be further assessed, although Fpr1 expression has been reported in murine astrocytes [87].

## 5. Roles of FPR1 in Neuroinflammation and Neurodegeneration

PRRs are a crucial first-line defense system expressed in innate immune cells, inducing an immune response to injury or infection. Activation of PRRs by pathogen associated molecular patterns (PAMPs) or damage associated molecular patterns (DAMPs) result in the upregulation of inflammatory mediators which act synergistically to help eliminate the cause of damage [88]. While Toll-like receptors are the most extensively studied PRRs [89,90], several others exist. These include NOD-like receptors, C-type lectin receptors and FPRs [88,91]. The role of FPR1 in responding to both DAMPs and PAMPs is widely appreciated within the periphery [91,92], although the importance of this receptor within the CNS parenchyma is becoming more apparent [77].

**Figure 1:**
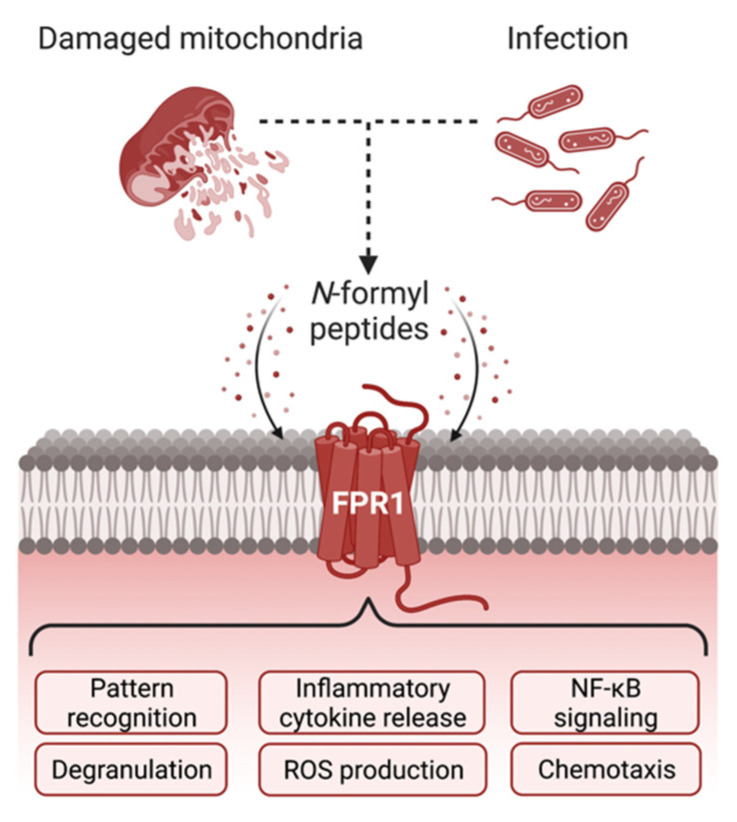
Cellular responses following FPR1 activation. Stimulation of FPR1 can be elicited by *N*-formyl peptides released from both invading bacterial pathogens and from damaged endogenous mitochondria. In the periphery, the resulting downstream signaling pathways within monocytes, macrophages and neutrophils help trigger a multifaceted immune response, including the release of inflammatory cytokines, reactive oxygen species, and the recruitment of additional immune cells via chemotaxis. Figure created with BioRender.com.

Conventionally, FPR1 ligands tend to be pro-inflammatory in effect, with activation of this receptor contributing to the induction of inflammatory responses [93,94]. Within the periphery, FPR1 has a central role in responding to infections through binding to bacterial and mitochondrial derived formyl peptides [27,69,95,96,97] (Figure 1). The depletion of FPR1 impairs neutrophil phagocytosis and killing of *E. coli* in vitro, and reduces neutrophil recruitment in vivo, a response associated with increased infection-induced mortality in mice [98,99]. In adults however, because the CNS is more frequently associated with sterile inflammation than infection, the importance of FPR1 in protection against CNS-associated infection remains to be clarified. Though, in pneumococcal-associated meningitis, a peripheral disease closely associated with the CNS, Fpr1 knockout increases both bacterial load and mortality rates in mice [99]. Further research on FPR1 expression and functioning in pathogenic models of encephalitis are needed to confirm its role(s) in CNS infection.

There is evidence that FPR1 may be important in sterile inflammatory responses within the CNS. In patients who displayed an intracerebral hemorrhagic injury, FPR1 mRNA and protein levels were both significantly upregulated compared to healthy controls, with the receptor being the most abundantly expressed PRR amongst those reported, more so than more classically studied Toll-like receptors 2 and 4, and the P2 × 4 purinoceptor [77]. These expression changes appeared to correlate with increased circulating mitochondrial *N*-formyl peptides post-hemorrhage, suggesting a feedback association between the agonists and their receptor [77]. The importance of FPR1 was confirmed in *Fpr1* knockout mice, where the acute CNS inflammatory profile was reduced, with final validation in wild-type mice treated with the Fpr1 antagonist T-0080, resulting in improved neurological outcomes and reduced oedema [77]. However, it was not determined if Fpr1 inhibition can improve mouse survival following experimentally induced hemorrhage. Future research is required to determine whether human FPR1 targeting may hold promise as a novel therapeutic strategy for intracerebral hemorrhage.

The role of FPR1 within the CNS does appear to vary depending on the original inflammatory insult, though, its importance in initiating the response to injury appears to be conserved across different models of disease. In a mouse model of traumatic brain injury, *Fpr1* knockout reduces tissue damage and acute neuroinflammation 24 h post-injury, but was associated with reduced neurogenesis 4-weeks post-injury [100]. The impact of these observations on animal survival outcomes was not assessed. In 12 month old APP/PS1 Alzheimer’s disease (AD) transgenic mice, mRNA transcripts and Fpr1 protein expression were upregulated [87]. Because of the poor prognostics of both AD and serious traumatic brain injury in humans, it is crucial to elucidate the precise roles of Fpr1 and its associated inflammatory pathways in these disease models.

## 6. Roles of FPR2 in Neuroinflammation and Neurodegeneration

**Figure 2:**
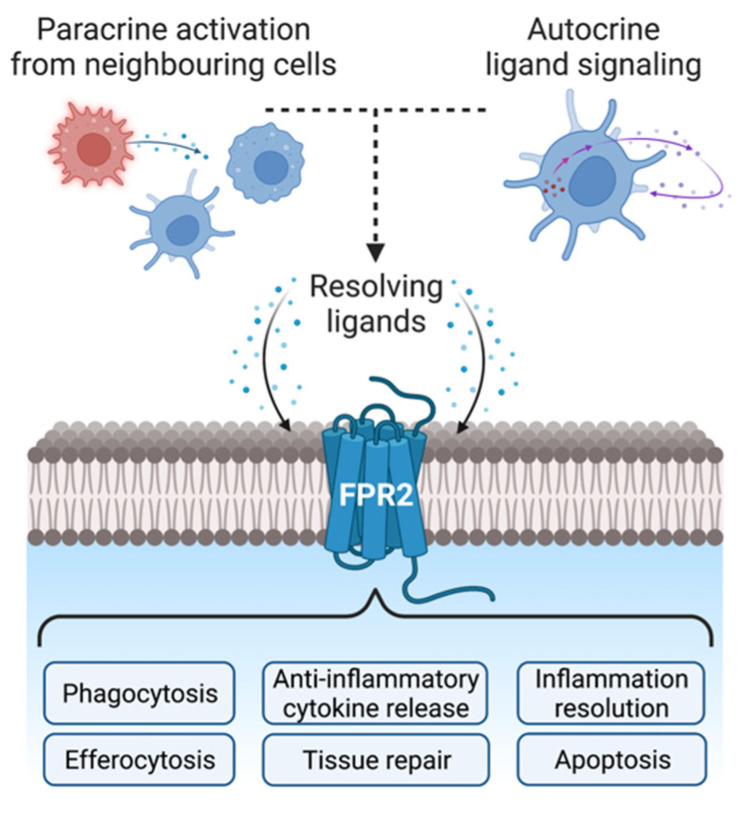
Cellular responses following FPR2 activation. There are several endogenous ligands that can stimulate FPR2, including the potent agonist annexin A1 (ANXA1). Cell types including macrophages, neutrophils and microglia can all participate in both paracrine and autocrine FPR2 signaling. Upon receptor activation, downstream signaling pathways elicit a broad inflammatory resolution response, including the release of anti-inflammatory cytokines, upregulating phagocytosis, removing damaged cells via efferocytosis, and contributing to tissue repair. Figure created with BioRender.com.

The importance of FPR2 for inflammatory resolution has become more evident in recent years (Figure 2), with many of its ligands reported as being anti-inflammatory, including small synthetic compounds such as the quinazolinone derivative Quin-C1, and the endogenously expressed protein Annexin A1 (ANXA1) [1,101,102]. Aligning with this extensive variety of ligands, FPR2 activation can elicit multiple different signaling pathways, although most of this work centers around ANXA1 [72,73]. Our group recently reported that ANXA1 can stimulate macrophage pro-resolving phenotypes via AMP-activated protein kinase (AMPK) phosphorylation, which contributed to murine muscle regeneration following injury [11]. Activation of extracellular signal-regulated kinases 1/2 (ERK1/2) and ETS transcription factor ELK1 (Elk1) by ANXA1 can also promote granulocyte differentiation and maturation from hematopoietic stem cells [103], while ANXA1 induced p38 mitogen-activated protein kinase (p38 MAPK) activation attenuates neuroinflammation following intracerebral hemorrhage in mice [104]. This signaling supports our previous findings, wherein we identified that ANXA1 can trigger p38 MAPK phosphorylation downstream from FPR2 [105,106].

The importance of FPR2 in resolving sterile peripheral inflammatory responses has been reported for many disease models, including promoting muscle fiber regeneration [11], alongside reducing diabetic nephropathy associated toxicity [107], inflammation associated cerebral thrombosis [108], acute experimental colitis [109] and arthritis [110]. While the extent of FPR2 function in response to sterile inflammation is still being mapped out for the CNS, support exists for a similar role to that observed within the periphery.

The role of FPR2 within the CNS, primarily through agonism by ANXA1 or lipoxin A_4_, has become more visible in recent years. Administration of ANXA1 following intracerebral hemorrhage injury in mice reduced microglial activation, brain oedema and acute neurological deficiencies, as determined with the sensorimotor Garcia testing paradigm [104]. However, the panel to determine microglial activation state was limited. At day 28 post-injury, spatial learning and memory was also improved in ANXA1 treated animals. Interestingly, ANXA1 has been reported to reduce thromboxane B_2_ and platelet function in both mice and humans, alongside promoting neutrophil elicited platelet phagocytosis [111]. Intravenous infusion of the ANXA1 *N*-terminal peptide Ac_2-26_ was also reported to shift microglia towards pro-resolving phenotypes at 3 days post-transient middle cerebral artery occlusion/reperfusion injury, highlighted by the reduction in pro-inflammatory (CD16, inducible nitric oxide synthase and IL-1β) and the increase in resolving markers (CD206, arginase-1, IL-10 and YM1), respectively [112]. These observations were reported in parallel with reductions in neuronal apoptosis and an increase in the integrity of the blood–brain barrier (BBB), the specialized vascular boundary consisting of brain microvascular endothelial cells [113]. In humans, the full ANXA1 protein was reduced by approximately 50% in the blood plasma of acute ischemic stroke patients compared to healthy controls in two separate cohorts [111,112], while restoration was possible following successful endovascular thrombectomy; a report which positively correlated to favorable clinical outcomes [112]. Thus, FPR2 modulation may hold therapeutic promise for ischemic and hemorrhagic associated CNS injury.

Further supporting protective roles of FPR2 in the CNS, ANXA1sp—a bioactive ANXA1 peptide—improved inflammatory profiles and neurological scores in a rat model of exsanguinating cardiac arrest [114]. Interestingly, increased protein expression of sirtuin-3 (SIRT3) and its downstream target forkhead box O-3 (FOXO3a) were also partially restored by ANXA1sp in this model. SIRT3 and FOXO3a are both associated with mammalian longevity and can counteract senescence induction in stem cells [115,116,117]. Several studies report that increasing the activity of these proteins could benefit neurodegenerative diseases such as AD [118,119,120], although this is somewhat debated for FOXO3 [121,122]. Thus, while a direct link between FPR2 activity and SIRT3 signaling is unknown, an interaction opens the possibility of FPR2 eliciting beneficial effects in a range of aging related diseases associated with cellular senescence.

There is also direct evidence to support a neuroprotective function of FPR2 in neurodegenerative disease, particularly in AD. Firstly, in 5XFAD AD transgenic mice, ANXA1 protein expression is reduced in both the brain and capillaries of the BBB [72,82]. Similar reductions were also observed in both the sera and brain of human AD patients. In vitro signaling analyses reported that ANXA1 stimulation of Fpr2 in immortalized murine BV2 microglia increases both the phagocytosis and degradation of toxic *A*β peptides [72]. The FPR2 agonist MR-39 also reduced fibrillary *A*β_1-42_ mediated proinflammatory cytokine release and increased an anti-inflammatory cytokine profile in organotypic hippocampal slice cultures (OHCs). Repeated intraperitoneal injections of MR-39 resulted in similar findings in 29-week old APP/PS1 transgenic mice, wherein neuronal apoptosis within the cortex and *A*β plaques in the hippocampus were both significantly reduced [123]. Interestingly, Trojan and colleagues also report that FPR2 inhibition with WRW_4_ was sufficient to prevent an increase in fibrillar *A*β_1-42_-induced IL-6 and TNFα in OHCs, although the statistical significance of the latter was not determined. Because *A*β is a known FPR2 ligand [12,124], this suggests that fibrillar *A*β_1-42_ aggregates can partially trigger inflammatory responses via FPR2 modulation. Additional studies have reported that pan-antagonism of FPR1/FPR2 with Boc-2 can also reduce neuronal *A*β pathology, increase the mRNA expression of several *A*β-degrading enzymes, decrease microglial ameboid morphology, and improve spatial memory in APP/PS1 transgenic mice [125]. Thus, modulating FPR2 not only with pro-resolving agonists, but also via selective blockade of *A*β interaction, may prove to be tactical research approaches in deciphering novel neuroprotective pathways in models of AD.

## 7. Expression Patterns of Endogenous FPR Ligand Annexin A1

As described above, FPRs are promiscuous receptors which interact with a wide range of ligands. Although many of these ligands have been chemically synthesized, such as Compound 43 and Quin-C1 [12], there are several key agonists which are endogenously expressed both in rodents and humans. While the primary FPR1 ligands (*N*-formyl peptides) are universally expressed in mitochondria and bacterial pathogens, several FPR2 ligands display specific endogenous expression patterns. Arguably one of the most important is the previously described ANXA1, a highly potent FPR2 agonist widely acknowledged to be involved in inflammatory resolution [11,126,127,128]. This robust pro-resolving FPR2 ligand is expressed in many eukaryotic species, but appears absent from both yeast and prokaryotes [129]. In particular, ANXA1 is highly expressed within cells and tissues associated with the immune response, commonly overlapping in distribution with FPR2, such as neutrophils, monocytes, macrophages and endothelial cells [11,130,131,132]. Localized in the cytosol, it can undergo plasma membrane translocation prior to cellular release and subsequent binding to, amongst other targets, FPR2 receptors expressed on the cell surface of neighboring or incoming cells [133]. While research into ANXA1 localization within the CNS is limited in comparison to the periphery, it is well documented to be highly expressed in endothelial cells of the BBB [130,134,135]. Microglial expression of ANXA1 is also consistently reported [128,134], although higher expression levels are likely correlated with active microglial phenotypes [72,136,137]. ANXA1 has also been reported in human astrocytes [138,139], although, similar to FPR2 expression, conflicting reports from the Human Protein Atlas and other research studies are apparent [140]. Interestingly, neuronal localized ANXA1 has been observed in murine dorsal root ganglia [84], hippocampal neurons [141,142], embryonic hypothalamic neurons [143], and retinal ganglion cells [144]. While ANXA1 nuclear translocation in neurons appears to be associated with apoptotic signaling following oxygen-glucose deprivation/reoxygenation injury [141,142,144], whether this function is independent of myeloid immunological mechanisms must be further clarified.

## 8. Considerations for Therapeutic Development

The formyl peptide receptor family are crucial pattern recognition receptors that respond to both infection and sterile inflammation. As such, these receptors are an attractive drug target for therapeutic interventions. The utilization of high throughput drug candidate screens [77], in tandem with proteomic methods, in vitro mechanistic research and in vivo disease modelling will provide a multipronged approach to determine the therapeutic potential of receptor ligands in protecting endogenous inflammatory pathways from pathological associated disruption. 

We have previously described the therapeutic promise of targeting the FPR system for neurodegenerative diseases, with a particular focus on FPR2 and AD [4]. However, the diverse binding capabilities of FPRs must be taken into consideration to minimalize significant hurdles in therapeutic research approaches. Firstly, while several molecules show select affinity towards one FPR, this is often negated by increased concentrations. For example, many ANXA1 N-terminal peptides are non-specific FPR1/FPR2 agonists [2], including Ac_1-25_, which can activate FPR1 at high concentrations, triggering pro-inflammatory signaling responses similar to traditional FPR1 agonists [145]. As such, the selectivity of any proposed novel FPR agonist will need to be validated with both genetic ablation and pharmacological inhibition of the FPRs in research models.

The ability for ligands to stimulate conformational changes in FPRs, facilitating both homo- and heterodimerization [106], may contribute to their astounding diversity. However, additional FPR dimerization research studies are not available to validate these initial findings. Interestingly, FPR2 can also interact with other receptors, including the receptor for advanced glycation end products (RAGE) in primary murine astrocytes, microglia and transfected HEK293 cells [87]. Receptor interactions between FPR2 and the scavenger receptor MARCO have also been reported in microglia [146,147]. While RAGE has been implicated in exacerbating *A*β pathology in AD models [148], a pathogenic role for MARCO is less clear. Yet, because *A*β displays agonism for both of these receptors [12,124], deciphering the consequences of ligand-induced interactions with FPR2 will be crucial in identifying new avenues for FPR2 therapeutic modulation.

While our knowledge of FPR1 and FPR2 signaling has improved in recent years, the physiological role of FPR3 remains relatively elusive. It was previously reported in HEK293T cells that the receptor displays a marked phosphorylation state under resting conditions compared to both FPR1 and FPR2 [149]. The subcellular localization also appeared unique, displaying interactions within intracellular vesicles prior to receptor stimulation, suggesting the receptor undergoes intrinsic endocytosis. Comparatively, FPR1/FPR2 only undergo vesicular endocytosis upon ligand binding, a characteristic feature for many GPCRs [150,151,152]. While current research is lacking, understanding the differences in receptor localization will be important to decipher the physiological role of FPR3, and whether its pathological modulation holds importance for the development of disease.

## 9. Overall Conclusion

Formyl peptide receptors are complex, multifunctional, and promiscuous receptors which display central roles in initiating, propagating, and resolving the inflammatory response. While most research has focused on their roles in infectious and sterile inflammation within the periphery, newer insights indicate their importance within the central nervous system. Thus, their responses to neuroinflammatory insults in neurodegenerative conditions cannot be overlooked. As such, future FPR research may be of critical importance in the development of neuroinflammatory-associated therapeutics.

## Figures and Tables

**Table 1:** Selective ligands of FPR1 and FPR2. Molecules are broadly grouped based on their structure and origins. Binding selectivity for each ligand has been provided. Available in vitro pK_D_, pEC_50_ and pIC_50_ values for ligand interactions with the human FPRs are included. Data has been adapted, compiled and condensed from previously available reviews [12,23,24]. n.d.; values not determined for the human receptors.

Ligand	Origin	Selectivity	pK_D_	pEC_50_	Model	Refs
**Formylated bacterial ligands**						
fMLF	*E. coli*	FPR1	6.4–9.3	4.6	Human neutrophils, L cells, RBL-2H3	[2,25,26]
fMIFL	*S. aureus*	FPR1, FPR2	n.d.	n.d.	Mouse neutrophils, RBL-2H3	[27,28]
fMIVTLF	*Listeria*	FPR1, FPR2	n.d.	n.d.	RBL-2H3	[28]
fMVMKFK	*Haemophilus*	FPR1, FPR2	n.d.	6.1, 8.1	HEK293	[29]
**Formylated mitochondrial ligands**						
fMLKLIV	Mitochondria	FPR1, FPR2	n.d.	7.4, 7.3	HL-60	[30]
fMMYALF	Mitochondria	FPR1, FPR2	n.d.	8.0, 7.8	HL-60, RBL-2H3	[28,30]
Mitocryptide-2	Mitochondria	FPR2	n.d.	6.2–6.4	Human neutrophils, HEK293T	[31,32]
**Non-formylated pathogen-derived ligands**						
C5a peptide	Hepatitis C virus	FPR2	n.d.	n.d.	Human monocytes and neutrophils, HEK293, RBL-2H3	[33]
gG-2p20	Herpes simplex virus	FPR1	n.d.	6.2–6.3	Human monocytes and neutrophils	[34]
Hp(2-20)	*Helicobacter pylori*	FPR2	n.d.	6.5	Human monocytes	[35]
**Non-mitochondrial host-derived ligands**						
Aβ	Host	FPR2	n.d.	7.0	Human monocytes, mouse neutrophils, HEK293, RBL-2H3	[36,37]
Annexin A1	Host	FPR2	6.5	n.d.	Human neutrophils, HEK293	[11,38,39,40]
Lipoxin A4	Host	FPR2	8.8–9.3	~12.0	Human neutrophils	[41,42,43,44]
Resolvin D1	Host	FPR2	~11.9	n.d.	Human neutrophils	[44]
Serum Amyloid A	Host	FPR2	n.d.	6.6–7.3	Human monocytes and neutrophils, HEK293	[45,46,47]
LL-37	Host	FPR2	n.d.	6.0	Human monocytes, neutrophils, and T cells, HEK293, RBL-2H3	[48,49]
**Natural peptide ligands**						
Cyclosporin H	*T. inflatum* & *T. polysporum*	FPR1	7.0 (pIC_50_)	n.d.	Human neutrophils	[50]
**Synthetic peptide ligands**						
Ac_9-25_	Synthetic	FPR1	n.d.	4.7	Human neutrophils, HL-60	[51]
Ac_2-26_	Synthetic	FPR1, FPR2	5.9	5.8–6.1	Human neutrophils, HEK293	[38,39,52]
WKYMVm	Synthetic	FPR2	10.1	n.d.	human neutrophils, HL-60	[53]
WRW_4_	Synthetic	FPR2	6.6 (pIC_50_)	n.d.	Human neutrophils, RBL-2H3	[54]
**Small molecule ligands**						
Compound 43	Synthetic	FPR1, FPR2	n.d.	n.d.	CHO, RBL-2H3	[28,55,56]
Compound 17b	Synthetic	FPR1, FPR2	n.d.	n.d.	CHO	[28,56]
Quin-C1	Synthetic	FPR2	n.d.	5.7–6.2	Human neutrophils, RBL-2H3	[28,57]
Quin-C7	Synthetic	FPR2	5.2 (pIC_50_)	n.d.	HeLa, RBL-2H3	[58]

## Data Availability

The Allan Brain Atlas can be accessed at www.brain-map.org. Data from the Human Brain Transcriptome Project can be accessed via www.hbatlas.org.
